# Ventricular Assist Device Research and Development in Brazil: A Long and Promising Relationship Between Medicine and Engineering

**DOI:** 10.21470/1678-9741-2023-0074

**Published:** 2023-09-11

**Authors:** Carlos J. T. Karigyo, Jeison W. G. Fonseca, André G. Boscarato, Mônica M. S. Matsumoto, Aron J. P. Andrade

**Affiliations:** 1 Centro de Engenharia em Assistência Circulatória, Instituto Dante Pazzanese de Cardiologia, São Paulo, São Paulo, Brazil; 2 Programa de Pós-graduação em Medicina/Tecnologia e Intervenção em Cardiologia, Universidade de São Paulo, São Paulo, São Paulo, Brazil; 3 Programa de Pós-graduação em Ciência Animal com Ênfase em Produtos Bioativos, Universidade Paranaense, Umuarama, Paraná, Brazil; 4 Clínica Cirúrgica de Grandes Animais, Hospital Veterinário, Universidade Paranaense, Umuarama, Paraná, Brazil; 5 Medical Electrical Devices Laboratory, Electronics Engineering Division, Instituto Tecnológico de Aeronáutica, São José dos Campos, São Paulo, Brazil

Heart transplantation (HTx) continues to be the gold standard therapy for advanced heart failure refractory to the conservative treatment. Nonetheless, HTx remains as a limited procedure in face of shortage of donors and poor medical conditions that many potential recipients carry or develop over the natural history of the disease. Because of these factors, several patients get their clinical status worsened at the transplant waiting list, and even though with a prioritization path, many of them deteriorate to an unfavorable scenario to receive a heart^[1]^.

The application of mechanical circulatory support devices aims to maintain alive and stable the patients who develop severe clinical conditions that could disable them from receiving a heart in a short term, serving as a bridge therapeutic strategy (bridge-to-transplantation [BTT])^[1]^. Historically, artificial pumps were developed to support and provide adequate perfusion for patients with difficult weaning from cardiopulmonary bypass (CPB) after heart operations, becoming useful for giving hemodynamic support to bridge transplant candidates when HTx era started in the 1960s. Briefly, left ventricular assist devices (LVADs) were composed by pulsatile pumps, evolving to implantable continuous-flow axial pumps and posteriorly to continuous-flow centrifugal pumps.

Currently, LVADs are an accepted therapy in many countries and commonly applied to a variety of heart diseases that lead to circulatory failure, serving not just for BTT strategy, but for bridge-to-decision, bridge-to-recovery, or for destination therapy^[2]^.

## Mechanical Circulatory Support in Brazil

The world first HTx was performed in 1967 by Christian Barnard, in South Africa. Six months later, Euryclides Zerbini performed the first HTx in Brazil. Around that same time, artificial pumps were just beginning to be developed and implanted as a measure to recover and wean the failing hearts from CPB after cardiac operations. In 1969, Denton Cooley, in the United States of America, implanted the first total artificial heart (TAH) in a patient who would be transplanted later, being considered the inauguration of BTT^[3]^.

Despite the Brazilian’s pioneering contribution in HTx and the traditional history of incorporating cutting-edge technologies in different fields of cardiac surgery, the routine application of mechanical assist devices is still a distant reality in our country. The application of such devices has been limited to support patients in post-cardiotomy shock, in most of the cases, using foreign manufactured devices as centrifugal pumps and oxygenators for extracorporeal membrane oxygenation circuits^[4]^.

The lack of financial support and funding from public and most private healthcare agencies obstructs the access of a vast majority of patients to this salvage treatment. A limited but successful number of experiences from patients bridged to transplantation with LVADs, especially in Chagas cardiomyopathy and in the pediatric population, showed improved survival and HTx rates^[4,5]^.

A few prominent researchers have been dedicated to overcoming the inherent challenges of clinical and translational research. In the state of São Paulo (Brazil), two major centers have been at the forefront of ventricular assist device (VAD) research and development. The Instituto do Coração of the Universidade de São Paulo released a paracorporeal pulsatile VAD currently in clinical use and conducts many other research projects. The Instituto Dante Pazzanese de Cardiologia holds a dedicated engineering center for VAD technology, being responsible for creating several prototypes and notable devices, like the first Brazilian TAH^[5,6]^.

## The Engineering Center for Circulatory Assistance

The Instituto Dante Pazzanese de Cardiologia was founded in 1954. In the 1960s, the institute achieved major pioneering contributions like the first national project of a CPB machine and a blood oxygenator, as well as the creation of the first implantable cardiac pacemaker and a prototype of an implantable pneumatic pump for ventricular assistance. In 2009, few decades later and with some new devices already developed, it was then created the Centro de Engenharia em Assistência Circulatória (CEAC) at the institute, the first center in Latin America exclusively dedicated to the development of blood pumps and devices for mechanical circulatory assistance^[6]^.

## The Auxiliary Total Artificial Heart

In the 1990s, important advances in the creation of devices for circulatory assistance were carried out. Initially, with collaborations from Dr. Yukihiko Nosé’s research team from Baylor College of Medicine, the Dante Pazzanese group proposed a new project of an artificial heart. But, differently from an orthotopically implanted device, they proposed a heterotopic artificial heart, the so-called auxiliary total artificial heart (ATAH). This new device was based on the same electromechanical principle as a TAH from Baylor College, but with innovative design features, technologies, and different applied materials^[6]^.

**Fig. 1:**
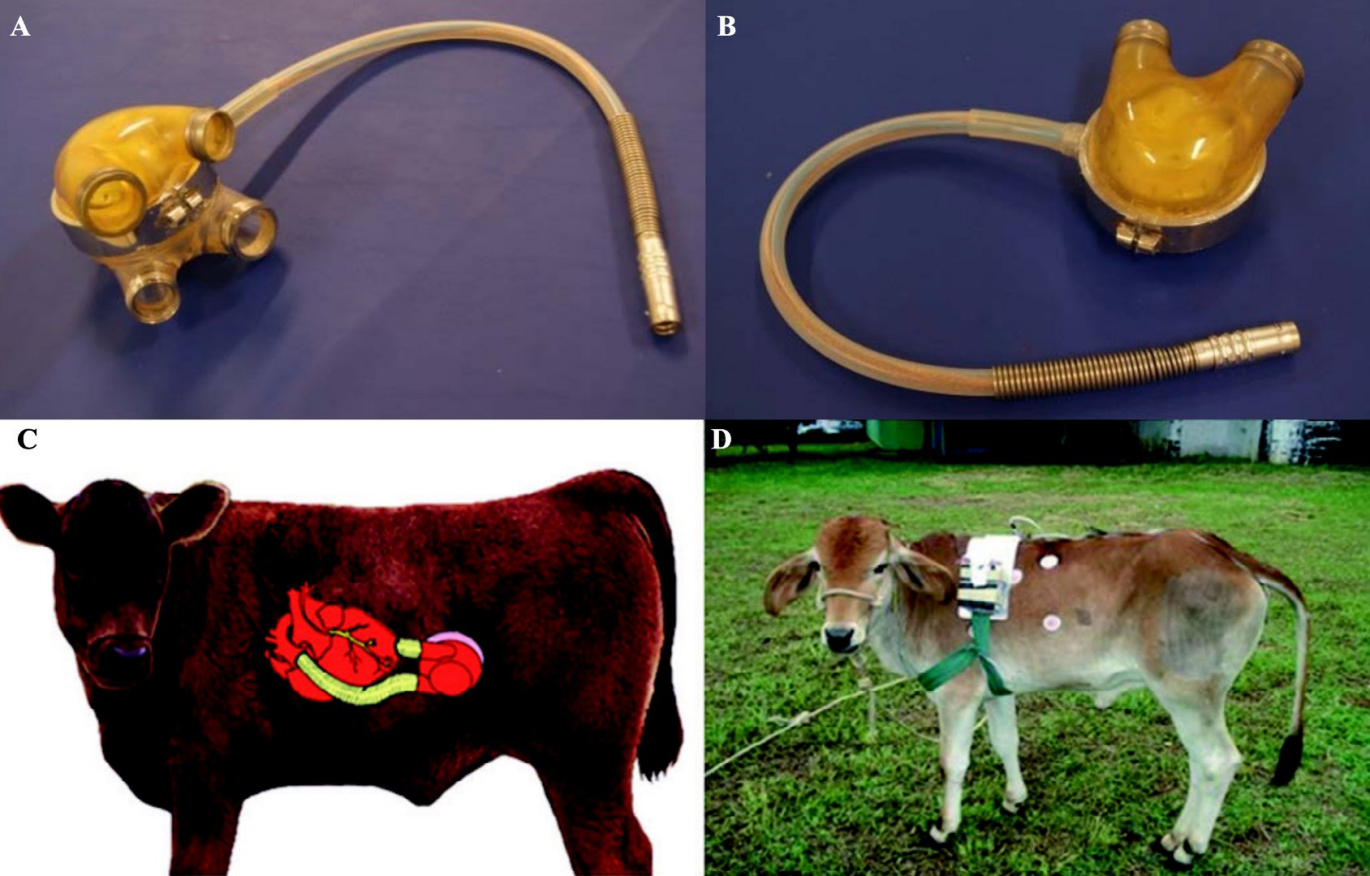
(A) The auxiliary total artificial heart configured as a biventricular assist device, being composed by two diaphragms housed in two pumping chambers; (B) configuration as univentricular assist device, being able to work as right ventricular assist device or left ventricular assist device (LVAD); (C) anatomical disposal of the implanted device as LVAD, and (D) calf in the recovery phase after device implant experimentation.

The ATAH works through an electromechanical mechanism that produces a pulsatile flow and is composed by two diaphragms housed in two pumping chambers. It can be applied as a univentricular or a biventricular VAD, as well as a TAH indeed, fully replacing the native organ (Figures 1A and 1B). But the original purpose of applying the ATAH was to support the native heart without removing it, providing an easier and faster implantability, safer conditions in case of device failures, and the possibility of reverse remodeling of the native heart^[7]^.

Between 1999 and 2009, preclinical studies with the ATAH were performed in calves (Figures 1C and 1D), with improvements in surgical techniques for implantation and device components^[8]^. In 2012, the ATAH became the first Brazilian TAH approved for clinical trials in the country^[6]^. However, the clinical trial didn’t move forward.

## New Centrifugal Pumps

**Fig. 2:**
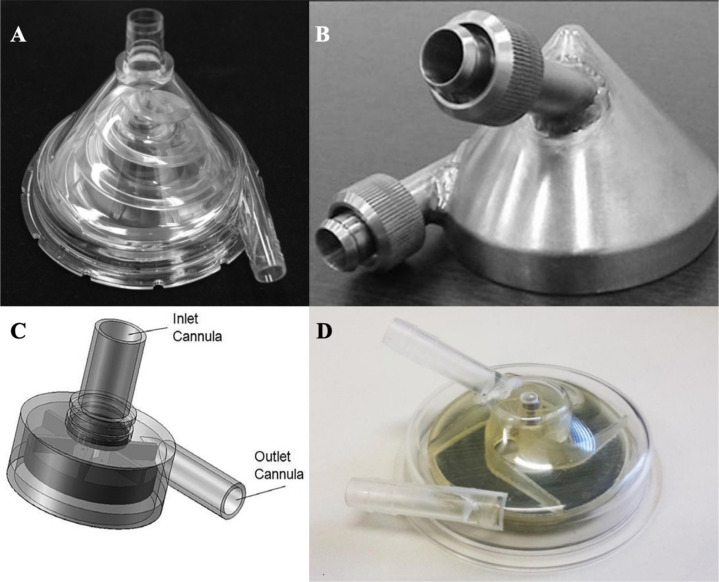
(A) Spiral Pump: combination of centrifugal and axial pumping principles with a conically shaped rotor, designed for cardiopulmonary bypass. (B) Implantable centrifugal blood pump: the first Brazilian centrifugal ventricular assist device (conically shaped), conceived to be fully implantable, with the pump being placed in the thoracic or abdominal cavity. (C) Apico aortic blood pump: a miniaturized centrifugal left ventricular assist device, with connections between inlet to left ventricular apex and outlet to ascending aorta, and the pump placement inside the pericardial cavity. (D) Temporary circulatory support device: new model of centrifugal blood pump for temporary ventricular assistance, with the purpose of application as a bridge-to-decision or a bridge-to-recovery strategy.

The CEAC of the Instituto Dante Pazzanese de Cardiologia developed other new models of blood pumps over the time. In the early 1990s, a new device started to be developed for CPB, combining centrifugal and axial pumping principles with a conically shaped rotor (Figure 2A), becoming known and later patented as the Spiral Pump (Fundação Adib Jatene, Brazil). The purpose was to provide a domestic and a more accessible product which could be used during cardiac surgeries^[6,9,10]^.

Following similar principles of design and operation of the Spiral Pump, but aiming intracorporeal implantation, a new blood pump was developed to work as a long-term LVAD. The implantable centrifugal blood pump was conceived in 2006, being considered the first Brazilian centrifugal VAD, consisting of a conical shape (Figure 2B), and producing an axial profile to the resulting flow^[11]^. The axial component of the blood flow proved to be advantageous by avoiding areas of stagnation inside the pump, thus preventing the formation of thrombi and providing greater assistance. The implant consisted of connecting the inlet cannula to the left ventricular (LV) apex and the outlet cannula to the descending aorta, with the pump being positioned inside the thoracic or abdominal cavity^[11,12]^.

In the early 2010s, based on similar models of third generation pumps available on the market, the CEAC created a new miniaturized centrifugal LVAD that could be implanted inside the pericardial cavity (Figure 2C). The apico aortic blood pump (AABP) is surgically connected between the LV apex and the ascending aorta through a polytetrafluoroethylene graft. The original purpose of the AABP was to provide circulatory assistance as a BTT strategy^[13]^. However, durability tests were favorable to an eventual application of the device as a long-term circulatory assistance or for destination therapy^[14]^.

More recently, a new model of centrifugal blood pump for temporary ventricular assistance has been developed and evaluated, with the purpose of application as a bridge-to-decision or a bridge-to-recovery strategy. Named as temporary circulatory support device (TCSD), it follows the principle of centrifugal pumping associated with the use of ceramic supports instead of bearings and without sealing gaskets (Figure 2D). In this way, the device has lower risks for thrombus formation, heating, and hemolysis, thus providing assistance for up to 30 days. The results of the hydrodynamic and hemolysis performance tests with the TCSD were satisfactory for the requirements necessary for temporary circulatory assistance, obtaining high hydrodynamic performance and low hemolysis rates^[15]^.

## Preclinical Studies in VAD Research and Development

The development of cardiovascular devices requires the use of simulators that faithfully reproduce the physiological parameters of the circulatory system, as well as the performance and interactions of such devices in different pathological conditions. Cardiovascular simulators can be classified as physical, computational, or hybrid (physical and computational)^[16]^. The adoption of hybrid models results from the advantage of these simulators to reproduce certain cardiovascular parameters more easily through computational algorithms, making experiments more sophisticated, adding complexity to the physical model^[17]^. The CEAC team created a new hybrid cardiovascular simulator with the aim of testing VADs in development^[16]^. With this novel test platform for ventricular simulation, several experiments and device improvements could be performed without the use of animal models.

Normally, *in vitro* studies precede *in vivo* experiments. The use of cardiovascular system simulators plays a key role by minimizing the use of animals in experiments. Nevertheless, the development of implantable devices must go through preclinical evaluations in animal models at some point of the process, since anatomical and physiological similarities allow procedures and techniques to be applied in large animal species as they would be applied in humans. There are some disadvantages though including higher costs of maintenance and the need for specialized surgical facilities and veterinary care, but the experiments with large animals in VAD research can provide translational data that overcome these issues. These studies have provided critical information for pump and cannula design, anatomical positioning, surgical techniques, performance and interactions to the devices, as well as for the development of new therapeutic modalities, like stem cell therapy with LVAD and improvements for destination therapy^[18-20]^.
